# Epithelial responses and *Candida albicans* pathogenicity are enhanced in the presence of oral streptococci

**DOI:** 10.1590/0103-6440202305420

**Published:** 2023-07-17

**Authors:** Loyse Martorano-Fernandes, Arella Cristina Muniz Brito, Elza Cristina Farias de Araújo, Leopoldina de Fátima Dantas de Almeida, Xiao-Qing Wei, David Wynne Williams, Yuri Wanderley Cavalcanti

**Affiliations:** 1 Graduate Program in Dentistry. Federal University of Paraíba. Cidade Universitária, João Pessoa, Paraiba, Brazil; 2 Department of Clinic and Social Dentistry. Federal University of Paraíba. Cidade Universitária, João Pessoa, Paraiba, Brazil; 3 Oral and Biomedical Sciences, School of Dentistry, Cardiff University, Cardiff , Wales, United Kingdom

**Keywords:** Biofilms, Interleukins, Pathogenicity, Candida albicans, Streptococcus

## Abstract

Experimental models that consider host-pathogen interactions are relevant for improving knowledge about oral candidiasis. The aim of this study was to assess the epithelial immune responses, *Candida* penetration of cell monolayers, and virulence during mixed species culture infections. Single species cultures of *Candida albicans* and mixed cultures (*C. albicans*, *Streptococcus mutans,* and *Streptococcus sanguinis*) were used to infect monolayers of HaCaT and FaDu ATCC HTB-43 cells for 12 h. After infection, IL-18 and IL-34 gene expression was measured to assess epithelial cell immune responses, and lactate dehydrogenase (LDH) activity was measured as an indicator of cell damage. Microscopy determined *C. albicans* morphology and penetration of fungal cells through the keratinocyte monolayer. Monolayers devoid of infection served as controls. Data were analyzed by an ANOVA one-way test followed by Tukey’s post-hoc test (α = 0.05). The results found that IL-18 and IL-34 gene expression and LDH activity were significantly (p < 0.05) upregulated for both cell lines exposed to mixed species cultures compared with *C. albicans* alone. *Candida albicans* yeast and hyphae were evident in *C. albicans* only infections. In contrast, monolayers infected by *C. albicans*, *S. mutans,* and *S. sanguinis* exhibited higher microbial invasion with several hyphal aggregates detected. The presence of streptococci in *C. albicans* infection enhances the virulence and pathogenicity of the fungus with associated increased immune responses and tissue damage. Extrapolation of these findings to oral infection would indicate the added potential benefit of managing bacterial components of biofilms during treatment.

## Resumo

O objetivo deste estudo foi avaliar a resposta epithelial imune, a colonização da *Candida albicans* em monocamadas celulares e sua virulência em resposta a infecções de culturas de biofilme multiespécie. Culturas de biofilme monoespécie de *C. albicans* e culturas mistas (C. albicans, *Streptococcus mutans e Streptococcus sanguinis)* foram utilizadas para infectar monocamadas de células HaCaT e FaDu por 12 h. Após a infecção, a expressão dos genes IL-18 e IL-34 foi medida para avaliar as respostas imunes das células epiteliais. A atividade da lactato desidrogenase (LDH) foi medida como um indicador de dano celular. A microscopia determinou a morfologia de *C. albicans* e a penetração das células fúngicas através da monocamada de queratinócitos. Monocamadas em que não houve infecção serviram como controles. Os dados foram analisados por um teste ANOVA one-way seguido pelo teste post-hoc de Tukey (α = 0,05). Os resultados demonstraram que a expressão gênica de IL-18 e IL-34 e a atividade de LDH foram (p < 0,05) reguladas positivamente para ambas as linhagens de células expostas a culturas de espécies mistas em comparação com *C. albicans* isoladamente. Leveduras de *C.albicans* e hifas foram evidentes em infecções apenas por *C. albicans*. Entretanto, monocamadas infectadas por *C. albicans, S. mutans e S. sanguinis* exibiram maior invasão microbiana com vários agregados de hifas detectados. Dessa maneira, a presença de estreptococos na infecção por *C. albicans* aumentou a virulência e a patogenicidade do fungo com respostas imunes aumentadas associadas a danos nos tecidos. A extrapolação desses achados para a infecção oral indicaria o potencial benéfico do controle dos componentes bacterianos em biofilmes durante a terapia da candidíase

## Introduction

*Candida albicans* is a commensal fungus that colonizes the mucosa and prosthetic devices in the oral cavity ^1^. Usually, *C. albicans* is a normal component of the microbial microbiome where it predominates in its yeast morphology, which has a lower potential for harmful effects on the host. However, often in cases of poor oral hygiene, or where there is host debilitation, *C. albicans* can form robust and complex biofilms ^2^. In such conditions, *C. albicans* exhibits higher yeast-to-hyphal (filamentous form) transition, with the latter having a greater association with pathogenicity. Hyphal forms of *C. albicans* can physically penetrate the oral epithelium and promote oral candidiasis ^2^ ^,^ ^3^ ^,^ ^4^.

During oral candidosis, *C. albicans* produces a range of virulence factors including phospholipases, secreted aspartyl proteinases, and toxins that can promote inflammatory responses ^5^ ^,^ ^6^. In such processes, epithelial cells are essential in the activation of signaling responses and thus ensuing immune defenses against infection ^7^. The production of the cytokines interleukin-18 (IL-18) and interleukin-34 (IL-34) are key components in these responses. IL-18 induces the production of interferon-gamma (IFN- ɣ) through the activation of T cells, while IL-34 leads to the expansion of macrophage numbers and their maturation ^8^ ^,^ ^9^. Interleukin responses are critical in the recruitment of other effector cells to the infection site ^10^ ^,^ ^11^. Essentially, epithelial cells are the frontline defense against *C. albicans* infection*.* Although the immune responses are largely understood during *C. albicans* tissue invasion ^12^, the role of IL-18 and IL-34 still needs further knowledge including its gene expression in the context of epithelial invasion.

Considering the complex polymicrobial biofilms and dynamic interactions in the oral environment and in oral candidosis, *C. albicans* is often found in association with oral bacteria ^13^. Previous investigations have shown that *Candida*-*Streptococcus* interactions increase the virulence and pathogenicity of *C. albicans* ^13^ ^,^ ^14^ ^,^ ^15^ ^,^ ^16^ ^,^ ^17^, contributing to the invasiveness of *C. albicans*. As such, along with the potentiation of hyphal invasion, consideration of host response in these interkingdom infections is warranted. Thus, an experimental model that considers both groups of microorganisms may improve our knowledge of oral candidosis and offer additional targeted therapies. This present investigation aimed to evaluate the *in vitro* epithelial cell immune response, damage, and fungal invasiveness of *Candida-Streptococcus* cultures. The study innovation includes a new point of view of polymicrobial interactions considering the epithelial invasion and response during oral candidosis.

## Materials and methods

### Experimental design

This *in vitro* study used monolayers of HaCaT or FaDu human cells to evaluate epithelial responses induced by mixed cultures comprised of *C. albicans* and *Streptococcus* species. Infection groups (n=8 for each group) were single *C. albicans* and mixed species of *C. albicans*, *Streptococcus mutans,* and *Streptococcus sanguinis*. In addition, non-infected controls of both cell lines were used. After 12 h infection, the epithelial responses measured were IL-18 and IL-34 gene expression and induced tissue damage as determined by lactate dehydrogenase (LDH) activity. In addition, *C. albicans* morphology and invasiveness were ascertained by light microscopy. 

### Microorganisms and growth conditions

*Candida albicans* ATCC 90028, *S. mutans* ATCC 25175 and *S. sanguinis* ATCC 10556 were used to form test cultures. *Candida albicans* was initially cultured on Sabouraud Dextrose Agar (SDA) (Oxoid, Basingstoke, United Kingdom), while *S. mutans* and *S. sanguinis* were grown on Blood Agar (blood agar base; Oxoid) supplemented with 5% (v/v) of defibrinated horse blood (TCS Biosciences, Buckingham, United Kingdom). Cells were then cultured aerobically in Brain Heart Infusion liquid medium (BHI; Oxoid) for 24 h at 37°C. Resulting cultures were centrifugated (3000 *g* for 5 min), gently washed (×2) in phosphate-buffered saline (PBS; pH 7.0), and resuspended in Modified Dulbecco Eagle Medium (DMEM; Life Technologies, Paisley, UK) supplemented with 10% (v/v) Fetal Bovine Serum (FBS; Life Technologies) and 50 mM glucose. Using a spectrophotometer (DiluPhotometer™; Implen, Westlake Village, CA, USA), cell density was adjusted to an OD_600_ (1 × 10^5^ CFU/ mL of *C. albicans* and OD_600_ 1 × 10^7^ CFU/ mL of streptococci cells) ^18^. 

### Cell culture conditions and monolayer infection

HaCaT and FaDu ATCC HTB-43 cells were used to form two different human cell monolayers. Cells were cultured in Dulbecco's Modified Eagle Medium (DMEM; Life Technologies, Paisley, UK) supplemented with 10% (v/v) Fetal Bovine Serum (FBS; Life Technologies) without antibiotics at 37°C and in 5 % CO_2_ until > 85% confluence was reached. HaCaT and FaDu were seeded, separately, in 24-well plates reaching a final concentration of 2 × 10^5^ cells/well and formed a monolayer. Infection was performed using the microbial cultures previously described following the infection groups (n=8 for each group), single *C. albicans,* and mixed species of *C. albicans*, *Streptococcus mutans,* and *Streptococcus sanguinis.* The microorganisms were added to the plate at the cell density adjusted to an OD_600_ (1 × 10^5^ CFU/ mL of *C. albicans* and OD_600_ 1 × 10^7^ CFU/ mL of streptococci cells) ^18^. Plates were incubated for 12 h at 37º C in a humidified 5% CO_2_ environment. After incubation, the supernatant and monolayer cells were collected for analysis. 

### Gene expression by qPCR

To determine immune epithelial response during infection, qPCR evaluated IL-18 and IL-34 gene expression. Primers were designed from full-length gene sequences obtained from the PubMed nucleotide platform using Primer3 software ^19^ (Table 1). GAPDH and β-actin served as reference genes (Table 1). 

Cell monolayers were collected and incubated in Trizol®, for 2 min. Extraction of total RNA was performed using the RNeasy Mini Kit (QIAGEN) in accordance with the manufacturer's instructions and following previous investigations (Cavalcanti et al., 2015). Reverse transcription reactions for cDNA synthesis used 5 μl of total RNA (200 ng μl^-1^) template, 1 μl of 10 mM dNTPs, 1 μl of 50 μg ml^-1^ random primers, 1 μl of 25 U μl^-1^ RNasin RNase inhibitor, 5 μl of M-MLV reaction buffer (×5) and 1 μl of 200 U μl^-1^ M-MLV (Promega, Southampton, UK). Molecular-grade water was added to achieve a final reaction volume of 25 μl. The reaction mix was incubated at 70°C for 5 min, followed by incubation at 37°C for 60 min. The resulting cDNA was stored at -20°C before use for qPCR.

**Table 1:** Forward (F) and reverse (R) primers used for evaluation of *C. albicans* virulence gene expression by quantitative polymerase chain reaction (qPCR)

Target gene	Sequence (5' ( 3')
IL-18	F - CCTTCCAGATCGCTTCCTCTCGCAACAA
Interleukin 18	R - CAAGCTTGCCAAAGTAATCTGATTCCAGGT
GAPDH	F - ACATCATCCCTGCCTCTAC
Housekeeping Gene	R - CCACCTTCTTGATGTCATCATATTTG
ß -Actin	F - GAGCACAGAGCCTCGCCTTTGCCGAT
Housekeeping Gene	R - ATCCTTCTGACCCATGCCCACCATCACG
IL-34	F - GGACAAGCTGCAGTACAGGAGCCGACTT
Interleukin 34	R - AGCCTGGTGACGTTGGCGATTCTGAACA

The qPCR was performed in 96-well plates in an ABI Prism 7000 instrument (Life Technologies) using a 20-μl reaction volume comprised of 2 μl of cDNA, 10 μl (×2) of SYBR-Green PCR Master Mix (Precision Master Mix; Primer Design, Southampton, UK), 1 μl of each primer (10 mM), and 6 μl of molecular biology grade water. An initial denaturation at 95°C for 2 min was undertaken, followed by 40 cycles of denaturation at 95°C for 15 s, primer annealing at 58°C for 30 s and primer extension at 72°C for 30 s. A final extension at 72°C for 2 min was performed, followed by cooling at 4° C. A primer dissociation stage at 60°C was used to generate a melting curve to verify the amplified product.

According to the amplification curves of genes evaluated, the threshold was adjusted. Based on the cycle number at which both the target and reference genes reached the threshold cycle (Ct), mean values of triplicate measurements were used to calculate the target gene’s expression using the ΔCt method ^20^.

### Lactate Dehydrogenase (LDH) activity

To measure human cell damage, the LDH activity was determined using an LDH Cytotoxicity Assay kit (Thermo Fisher Scientific, Cramlington, UK), following the manufacturer's instructions. After incubation, the cell culture supernatant was collected, added to the LDH enzyme assay reagent, and the absorbance read at 490/680 nm. Control samples were used for normalization ^18^. 

### Visualization of *C. albicans* invasiveness by inverted light microscopy

An inverted light microscope (Olympus CK2; Olympus Optical Go Ltd, London, UK). was used to visualize the proliferation of the microorganisms through HaCaT keratinocytes monolayers. Images were obtained using ×40 objective magnification and were qualitatively analyzed. 

### Statistical analysis

Statistical analysis was performed using the Statistical Package for the Social Sciences (SPSS, IBM, Chicago) with a significance of 5%. The assumptions for equality of variances and normal error distribution were assessed for each variable accordingly. One-way ANOVA was used to analyze epithelial cell gene expression and LDH activity. Post-hoc comparisons were performed using Tukey’s test.

## Results

**Figure 1:**
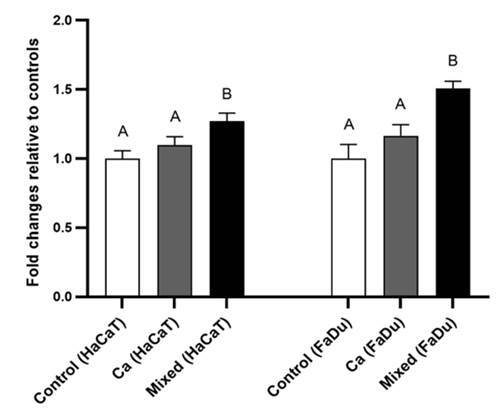
IL-18 gene expression (qPCR) for HaCaT and FaDu cell lines. Monolayers without infection (control); single *C. albicans* infection; mixed infection by *C. albicans, S. mutans,* and *S. sanguinis* (Ca + Sm + Ss). Human target genes were normalized using the ß-actin and GAPDH (housekeeping) genes. Different letters indicate a significant difference (p< 0.05).

**Figure 2:**
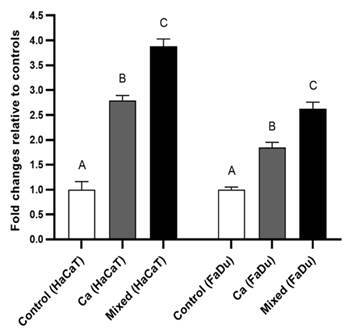
IL-34 gene expression (qPCR) for HaCaT and FaDu cell lines. Monolayers without infection (control); single *C. albicans* infection; mixed infection by *C. albicans, S. mutans,* and *S. sanguinis* (Ca + Sm + Ss). Human target genes were normalised using the ß-actin and GAPDH (housekeeping) genes. Different letters indicate a significant difference (p< 0.05).

Gene expression analyses showed that IL-18 and IL-34 genes were significantly (p < 0.05) upregulated for both cell lines when exposed to mixed species infections. In contrast, IL-18 gene expression was similar for uninfected cell lines and those exposed only to *C. albicans* (p > 0.05) (Figure 1). Gene expression of IL-34 was significantly (p < 0.05) elevated for cell lines infected with *C. albicans* compared with non-infected cell lines (Figure 2). 

**Figure 3:**
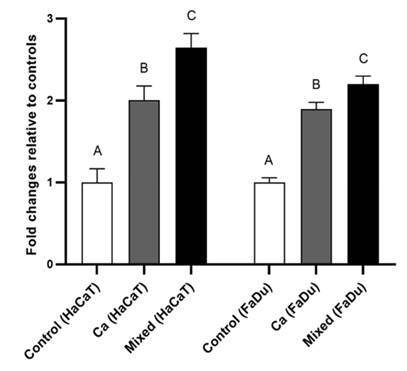
Lactate dehydrogenase (LDH) activity for HaCaT and FaDu cell lines. Monolayers without infection (control); single *C. albicans* infection; mixed infection by *C. albicans, S. mutans,* and *S. sanguinis* (Ca + Sm + Ss). Different letters indicate a significant difference (p< 0.05).

Based on LHD activity, tissue damage was significantly (p < 0.05) higher in cells infected by a mixed species inoculum compared with *C. albicans* only and non-infected tissue. Additionally, a statistical difference in LDH activity was found between *C. albicans* cell line infection and non-infected controls (p < 0.05) (Figure 3). 

**Figure 4:**
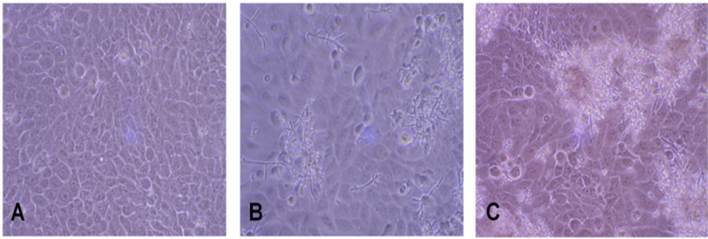
Representative images of keratinocytes monolayer and *C. albicans* morphology in the inverted light microscopy. A) Monolayers without infection (control). B) single *C. albicans* biofilms. C) mixed infection by *C. albicans, S. mutans,* and *S. sanguinis.* Note, in the single biofilm *C. albicans* yeast, and some hyphae dispersed. In mixed biofilms, intense microbial proliferation, and hyphae aggregates.

Representative images of keratinocyte monolayers and *C. albicans* morphology revealed microbial proliferation and *C. albicans* morphological state. In *C. albicans* only infections, *C. albicans* yeast, and some dispersed hyphae were evident. Interestingly, in monolayers infected by *C. albicans*, *S. mutans,* and *S. sanguinis*, higher microbial proliferation, and invasiveness were observed, with more numerous hyphal aggregates (Figure 4). 

## Discussion

The oral epithelium is the primary barrier to preventing microbial infection in the mouth ^7^. Therefore, understanding how epithelial cells respond to a fungi-bacterial infection would be beneficial and could be an indicator of the effectiveness of new therapeutic approaches. This study used HaCaT and FaDu cell lines. The former was used to investigate the response of keratinocytes (most abundant cells in oral mucosa), whilst FaDu cells have previously been reported as being less susceptible to damage by *C. albicans* ^21^ ^,^ ^22^. Also, we justify the chosen experimental in agreement with the period that the monolayers can stand before cell damage ^22^ ^,^ ^23^. Our findings revealed that a mixed *Candida*-*Streptococcus* infection increased the immune response, cell damage, and microbial proliferation and led to higher invasiveness by *C. albicans*. 

Cytokines released by epithelial cells may orchestrate the oral mucosa's proinflammatory process ^22^. Here, IL-18 and IL-34 genes were upregulated in both cell lines exposed to mixed biofilms. IL-18 is the so-called gamma interferon (IFN-γ) inducing factor ^11^ ^,^ ^24^. This interleukin is released by macrophages stimulating IFN‐γ production, which plays a role in host defense against *C. albicans* ^25^ ^,^ ^26^. In the present study, *C. albicans* infection did not increase IL-18 gene expression. This result contrasts with observations in murine models with disseminated candidiasis ^26^ ^,^ ^27^. Such differences in IL-18 production could relate to the time of infection, *C. albicans* morphology, or other differences between the models used. Importantly, when *Candida*-*Streptococcus* mixed biofilms were present, the IL-18 response was upregulated. These findings indicate that interaction between these species infections leads to increased fungal virulence with associated earlier proinflammatory responses. Importantly, a previous study with *Streptococcus sanguinis, Streptococcus mutans, Actinomyces viscosus,* and *Actinomyces odontolyticus* showed that bacterial-only infection did not promote significant tissue damage or invasiveness ^18^.

IL-34 gene expression was upregulated in HaCaT and FaDu-infected cells. Infection with *C. albicans*, *S. mutans,* and *S. sanguinis* led to higher IL-34 gene expression compared with *C. albicans* only. These findings reinforce our hypothesis that mixed-species infections elevate the ensuing immune response. IL-34 is suggested as a biomarker for infectious diseases and screening of anti-inflammatory therapies ^10^. This role has been assigned to IL-34 as this interleukin can promote tolerance to *C. albicans* through the downregulation of TLR2 and Dectin-1 expression by macrophages, resulting in lower TNFα production ^28^. Although IL-34 is important, only one investigation has evaluated its expression in *C. albicans* infection models ^28^. To our knowledge, this study is the first that explores IL-34 expression during *Candida*-*Streptococcus* interaction. 

In addition to the effect of bacterial presence on the epithelial immune response, tissue damage, and pathogenicity were also investigated. LDH activity is widely used to measure tissue injury during infection. Our findings revealed that *Candida*-*Streptococcus* infection promoted significantly higher damage of cells compared with those infected with *C. albicans* only. This result is in agreement with a previous investigation that explored interactions between *C. albicans* and *Streptococcus* species, where the presence of polymicrobial biofilms on Reconstituted human oral epithelium (RHOE), also resulted in higher damage, based on LDH ^18^. Polymicrobial interactions establish homeostasis and dysbiosis in the oral biofilm ^29^. Due to variations in microbial numbers or the host environment, microbial communities may interact to potentiate pathogenicity ^30^. For *Candida*-*Streptococcus* interactions, an increase in the capacity of *S. mutans* to demineralize the enamel of teeth has been reported ^14^ ^,^ ^31^. 

Similarly, interactions between *C. albicans* and *S. sanguinis* have been implicated in promoting an increase in the biofilm biomass to form robust biofilms ^32^. Finally, the presence of certain bacterial species significantly increases *C. albicans* virulence ^18^ ^,^ ^33^. It is important to highlight that other studies have been exploring the interactions between *Candida*-*Streptococcus* by the use of probiotics point of view, due to their ability to inhibit some virulence factors such as coaggregation and dimorphism ^34^ ^,^ ^35^, however, these comparisons should not be extrapolated because in the context of probiotics, *S. salivarius* K12, for example is a different reference strain, which can modulate different response when compared to those used in the present study. 

Also, in the present study, the morphological transition between yeast to hyphal forms was explored as a virulence factor ^36^. Microscopy demonstrated intense microbial proliferation with several hyphal aggregates after contamination with different species. Even though hyphal morphogenesis is a relevant virulence marker, this approach does not explore the mechanisms involved and this is an area for further study. The expression of a subset of genes encoding virulence factors (*e.g.* Hwp1, Als3, Sap4, Sap5, Sap6, Ece1, Hyr1 ^37^ would be relevant to further enhance our understanding of the mechanistic basis of bacterial-induced modulation of *C. albicans* virulence.

Although this study did not use single *S. mutans* and *S. sanguinis* infections as control, previous studies demonstrated that a bacterial biofilm of these species did not modulate host interleukins' gene expression ^18^. Moreover, the bacterial infections did not affect the LDH activity and had limited invasion and tissue proliferation compared to uninfected controls ^18^. Thus, these previous data support our findings that epithelial immune responses and pathogenicity of *Candida albicans* are enhanced in the presence of oral streptococci. 

Microscopy revealed higher *C. albicans* invasion of cell monolayers in mixed infections. *Candida albicans* can induce endocytosis or actively penetrate host cells ^36^. In the first process, the fungus expresses invasins (*i.e*. Als3 and Ssa1) which bind to host ligands, such as E-cadherin, triggering fungal entry into the host cell ^37^. In contrast, active penetration occurs by physical pressure exerted by directed hyphal growth, hyphal extension, and hydrolytic enzyme secretion ^38^. Despite the fact that these mechanisms are well established in single *C. albicans* biofilms, the bacterial contribution to endocytosis and active penetration remains poorly explored. 

Taken together, our results demonstrated an important contribution of oral streptococci to the epithelial immune response and pathogenicity of *C. albicans* infections. Clinically, consideration of the bacterial component as a potential modulator of candidosis might be of benefit in the management strategies of the infection.
